# Genome analyses of the carboxydotrophic sulfate-reducers *Desulfotomaculum nigrificans* and *Desulfotomaculum carboxydivorans* and reclassification of *Desulfotomaculum caboxydivorans* as a later synonym of *Desulfotomaculum nigrificans*

**DOI:** 10.4056/sigs.4718645

**Published:** 2014-03-01

**Authors:** Michael Visser, Sofiya N. Parshina, Joana I. Alves, Diana Z. Sousa, Inês A. C. Pereira, Gerard Muyzer, Jan Kuever, Alexander V. Lebedinsky, Jasper J. Koehorst, Petra Worm, Caroline M. Plugge, Peter J. Schaap, Lynne A. Goodwin, Alla Lapidus, Nikos C. Kyrpides, Janine C. Detter, Tanja Woyke, Patrick Chain, Karen W. Davenport, Stefan Spring, Manfred Rohde, Hans Peter Klenk, Alfons J.M. Stams

**Affiliations:** 1 Laboratory of Microbiology, Wageningen University, Wageningen, The Netherlands; 2 Wingradsky Institute of Microbiology, Russian Academy of Sciences, Moscow, Russia; 3 Centre of Biological Engineering, University of Minho, Braga, Portugal; 4 Instituto de Tecnologia Quimica e Biologica, Universidade Nova de Lisboa, Oeiras, Portugal; 5 Department of Aquatic Microbiology, Institute for Biodiversity and Ecosystem Dynamics, University of Amsterdam, Amsterdam, The Netherlands; 6 Department of Microbiology, Bremen Institute for Materials Testing, Bremen, Germany; 7 Laboratory of Systems and Synthetic Biology, Wageningen University, Wageningen, The Netherlands; 8 DOE Joint Genome Institute, Walnut Creek, California, USA; 9 Los Alamos National Laboratory, Bioscience Division, Los Alamos, New Mexico, USA; 10 Theodosius Dobzhansky Center for Genome Bionformatics, St. Petersburg State University, St. Petersburg, Russia; 11 Algorithmic Biology Lab, St. Petersburg Academic University, St. Petersburg, Russia; 12 Leibniz Institute DSMZ - German Collection of Microorganisms and Cell Cultures, Braunschweig, Germany; 13 HZI – Helmholtz Centre for Infection Research, Braunschweig, Germany

**Keywords:** Thermophilic spore-forming anaerobes, sulfate reduction, carboxydotrophic, *Peptococcaceae*, *Clostridiales*

## Abstract

*Desulfotomaculum nigrificans* and *D. carboxydivorans* are moderately thermophilic members of the polyphyletic spore-forming genus *Desulfotomaculum* in the family *Peptococcaceae*. They are phylogenetically very closely related and belong to ‘subgroup a’ of the *Desulfotomaculum* cluster 1. *D. nigrificans* and *D. carboxydivorans* have a similar growth substrate spectrum; they can grow with glucose and fructose as electron donors in the presence of sulfate. Additionally, both species are able to ferment fructose, although fermentation of glucose is only reported for *D. carboxydivorans*. *D. nigrificans* is able to grow with 20% carbon monoxide (CO) coupled to sulfate reduction, while *D. carboxydivorans* can grow at 100% CO with and without sulfate. Hydrogen is produced during growth with CO by *D. carboxydivorans*. Here we present a summary of the features of *D. nigrificans* and *D. carboxydivorans* together with the description of the complete genome sequencing and annotation of both strains. Moreover, we compared the genomes of both strains to reveal their differences. This comparison led us to propose a reclassification of *D. carboxydivorans* as a later heterotypic synonym of *D. nigrificans*.

## Introduction

In 1965, the genus *Desulfotomaculum* was created for sulfate-reducing bacteria that form heat-resistant spores [1]. One of the first species that was included in this new genus was *D. nigrificans* Delft 74, which was originally described as “*Clostridium nigrificans”* by Werkman and Weaver (1927) [2]. Later, Starkey (1938) renamed it to “*Sporovibrio desulfuricans”* [3] before it was finally renamed as *D. nigrificans* [1]. *D. nigrificans* is a moderate thermophile that typically grows with fructose and glucose coupled to sulfate reduction [1,4]; without sulfate, only growth with fructose was observed. Utilizing sugars is rare among *Desulfotomaculum* species. Additionally, *D. nigrificans* was described to be able to grow with a number of other substrates including lactate, ethanol, alanine, formate, and carbon monoxide (20%) coupled to sulfate reduction [5,6].

Another moderately thermophilic *Desulfotomaculum* species that can grow with glucose and CO is *D. carboxydivorans* CO-1-SRB [6]. *D. carboxydivorans* was isolated from sludge in an anaerobic bioreactor treating paper mill wastewater [6] and was described to be the first sulfate-reducing bacterium able to grow at 100% CO. *D. carboxydivorans* converted CO in the presence and absence of sulfate and produced hydrogen during CO conversion. *D. carboxydivorans* can also grow with glucose. In contrast to *D. nigrificans*, *D. carboxydivorans* degrades glucose both with and without sulfate.

Phylogenetically, *D. carboxydivorans* is most closely related to *D. nigrificans*. However, *D. nigrificans* is not able to produce hydrogen from CO. Therefore, by comparing the genomes of these strains, the physiological differences might be explained. Here we present a summary of the features of *D. nigrificans* and *D. carboxydivorans*, together with the description of the complete genome sequencing and annotation of both strains. Moreover, we compared the genomes of both strains to reveal differences between these phylogenetically very closely related strains. This comparison led us to propose to that *D. carboxydivorans* is a later heterotypic synonym of *D. nigrificans*.

### Classification and features

**Figure 1:**
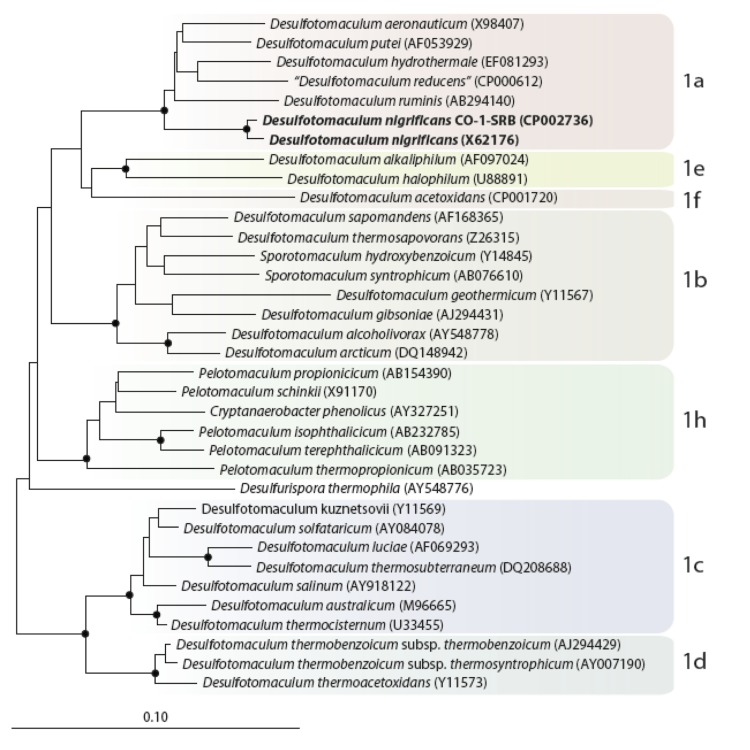
Neighbor joining tree based on 16S rRNA sequences showing the phylogenetic affiliation of *Desulfotomaculum* and related species divided in the subgroups of *Desulfotomaculum* cluster 1. DSM 574 and DSM 14880 are in bold type. The sequences of different *Thermotogales* were used as outgroup, but were pruned from the tree. Closed circles represent bootstrap values between 75 and 100%. The scale bar represents 10% sequence divergence.

Comparison of the 16S rRNA gene sequences of *D. carboxydivorans* CO-1-SRB DSM 14880 and *D. nigrificans* DSM 574 revealed that the two bacteria are highly related (99% sequence similarity). Both strains are part of the *Desulfotomaculum* cluster 1 subgroup a, together with *D. aeronauticum*, *D. putei*, *D. hydrothermale*, “*D. reducens*” and *D. ruminis* (Figure 1).

**Figure 2:**
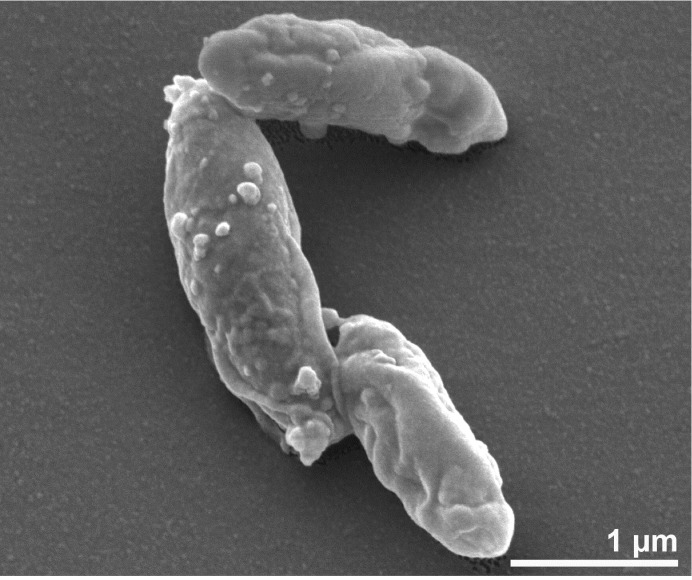
Scanning electron microscopic photograph of DSM 574

**Figure 3:**
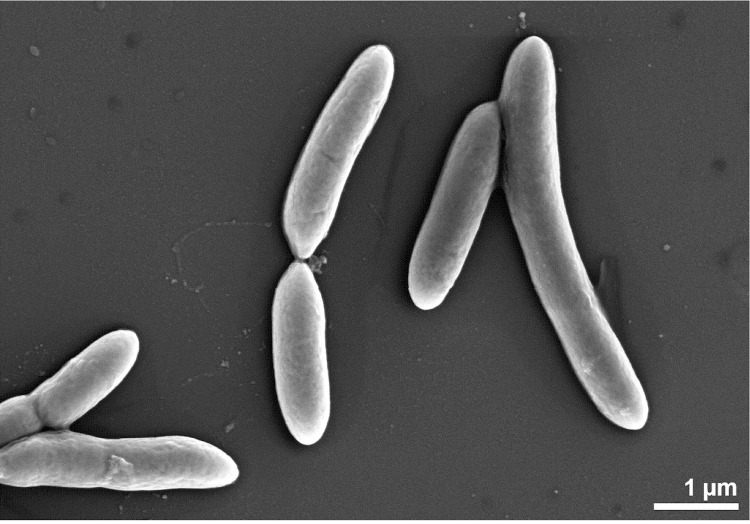
Scanning electron microscopic photograph of DSM 14880

*D. nigrificans* and *D. carboxydivorans* are Gram-positive, sulfate-reducing, rod shaped bacteria with rounded ends (0.3-0.5 μm thick and 3-6 μm long [1]; 0.5-1.5 μm thick and 5-15 μm long [6], respectively) (Figure 2 and Figure 3). They have a similar temperature range for growth and can both grow optimally at 55°C. Additional similarities can be found in the substrates used for growth. Both *D. nigrificans* and *D. carboxydivorans* can grow with fructose, glucose and alanine. These substrates are incompletely oxidized to acetate, coupled to sulfate reduction. Other suitable electron acceptors in addition to sulfate are thiosulfate and sulfite. Neither nitrate nor elemental sulfur are used as electron acceptors.

In the absence of an electron acceptor, *D. nigrificans* is able to grow by fermentation of fructose and pyruvate [7]. Additionally, *D. nigrificans* has been reported to grow with lactate and ethanol in syntrophic interaction with *Methanobacterium thermoautotrophicum* [5]. Syntrophic growth of *D. carboxydivorans* has never been tested. *D. carboxydivorans* is able to grow in the absence of an electron acceptor with CO (100%), pyruvate, lactate, glucose and fructose [6]. The cellular fatty acid patterns of the two strains were analyzed by Parshina et al. [6] and Krishnamurthi et al. [8]. Both fatty acid patterns are similar and the dominating fatty acids were identified as 16:0, iso 15:0, iso 17:0, anteiso 15:0, 18:0 and iso 16:0. Collins and Widdel [9] analyzed the respiratory lipoquinone content of *D. nigrificans* DSM 574 and found MK7 as the predominant isoprenoid quinone. A summary of the classification and general features of *D. nigrificans* and *D. carboxydivorans* is presented in Table 1 and 2, respectively.

**Table 1:** Classification and general features of *D. nigrificans* DSM 574 according to the MIGS recommendations [10]

MIGS ID	Property	Term	Evidence code^a^
	Current classification	Domain *Bacteria* Phylum *Firmicutes* Class *Clostridia* Order *Clostridiales* Family *Peptococcaceae* Genus *Desulfotomaculum* Species *Desulfotomaculum nigrificans* Type strain Delft 74	TAS [11] TAS [12-14] TAS [15,16] TAS [17,18] TAS [17,19] TAS [17,20,21] TAS [17,20]
	Gram stain	negative, with a Gram-positive cell wall structure	
	Cell shape	rods, rounded ends, sometimes paired	TAS [1]
	Motility	Slight tumbling, peritrichous flagella	TAS [1]
	Sporulation	oval, terminal or subterminal, slightly swelling the cell	TAS [1]
	Temperature range	30-70 °C	TAS [1]
	Optimum temperature	55 °C	TAS [1]
	Carbon source	glucose and other carbohydrates	TAS [1,4,5]
	Energy source	heterotrophic	TAS [1,4,5]
	Electron acceptor	sulfate, thiosulfate and sulfite.	TAS [4]
MIGS-6	Habitat	soils, compost heaps, thermal spring water, spoiled foods.	TAS [1]
MIGS-6.3	Salinity	not reported	
MIGS-22	Oxygen	obligate anaerobic	TAS [1]
MIGS-15	Biotic relationship	free living	TAS [1]
MIGS-14	Pathogenicity	none	TAS [1]
MIGS-4	Geographic location	Delft, The Netherlands	
MIGS-5	Sample collection time		
MIGS-4.1	Latitude	52.011	
MIGS-4.2	Longitude	4.360	
MIGS-4.3	Depth	not reported	

Evidence codes - TAS: Traceable Author Statement (i.e., a direct report exists in the literature); NAS: Non-traceable Author Statement (i.e., not directly observed for the living, isolated sample, but based on a generally accepted property for the species, or anecdotal evidence). Evidence codes are from the Gene Ontology project [22].

**Table 2:** Classification and general features of *D. carboxydivorans* DSM 14880 according to the MIGS recommendations [10]

MIGS ID	Property	Term	Evidence code^a^
	Current classification	Domain *Bacteria* Phylum *Firmicutes* Class *Clostridia* Order *Clostridiales* Family *Peptococcaceae* Genus *Desulfotomaculum* Species *Desulfotomaculum carboxydivorans* Type strain CO-1-SRB	TAS [11] TAS [12-14] TAS [15,16] TAS [17,18] TAS [17,19] TAS [17,20,21] TAS [17,20]
	Gram stain	negative, with a Gram-positive cell wall structure	TAS [6]
	Cell shape	rods, rounded ends, sometimes paired.	TAS [6]
	Motility	twisting and tumbling motion	TAS [6]
	Sporulation	oval, terminal or subterminal	TAS [6]
	Temperature range	30-68°C	TAS [6]
	Optimum temperature	55°C	TAS [6]
	Carbon source	100% CO, with and without sulfate	TAS [6]
	Energy source	hydrogenogenic and heterotrophic growth	TAS [6]
	Electron acceptor	sulfate, thiosulfate and sulfite.	TAS [6]
MIGS-6	Habitat	Paper mill waste water sludge	
MIGS-6.3	Salinity	0-17 g NaCl l^-1^	TAS [6]
MIGS-22	Oxygen	obligate anaerobe	TAS [6]
MIGS-15	Biotic relationship	free living	TAS [6]
MIGS-14	Pathogenicity	none	
MIGS-4	Geographic location	Eerbeek, the Netherlands	TAS [6]
MIGS-5	Sample collection time	1999	TAS [6]
MIGS-4.1	Latitude	52.104217	TAS [6]
MIGS-4.2	Longitude	6.060133	TAS [6]
MIGS-4.3	Depth	not reported	

Evidence codes - TAS: Traceable Author Statement (i.e., a direct report exists in the literature); NAS: Non-traceable Author Statement (i.e., not directly observed for the living, isolated sample, but based on a generally accepted property for the species, or anecdotal evidence). Evidence codes are from the Gene Ontology project [22].

## Genome sequencing and annotation

### Genome project history

*D. nigrificans* and *D. carboxydivorans* were selected for sequencing in the DOE Joint Genome Institute Community Sequencing Program 2009, proposal 300132_795700 'Exploring the genetic and physiological diversity of *Desulfotomaculum* species'. They are important for their position in subgroup a of the *Desulfotomaculum* cluster 1. Sequencing the complete genome of the two strains was proposed as it would allow the study of the genetic and physiological diversity within subgroup a. Furthermore, a comparison of the two genomes should reveal the genes involved in CO metabolism and the H_2_ production in *D. carboxydivorans*. The genome projects of *D. nigrificans* and *D. carboxydivorans* are listed in the Genome OnLine Database (GOLD) [23] as project Gi03933 and Gc01783, respectively. The two complete genome sequences were deposited in Genbank. Sequencing, finishing and annotation of the two genomes were performed by the DOE Joint Genome Institute (JGI). A summary of the project information of *D. nigrificans* and *D. carboxydivorans* is shown in Table 3.

**Table 3:** Genome sequencing project information of DSM 574 and DSM 14880.

**MIGS ID**	**Property**	**Term** (for DSM 574)	**Term** (for DSM 14880)
MIGS-31	Finishing quality	Permanent draft	Finished
MIGS-28	Libraries used	Three genomic libraries: 454 standard library, 454 PE libraries (7kb insert size), one Illumina library	Four genomic libraries: one 454 pyrosequence standard library, two 454 PE libraries (4kb and 11 kb insert size), one Illumina library
MIGS-29	Sequencing platforms	Illumina GAii, 454 GS FLX Titanium	Illumina GAii, 454 GS FLX Titanium
MIGS-31.2	Fold coverage	462.8 × Illumina; 35.2 × pyrosequence	116.8 × Illumina; 50.6 × pyrosequence
MIGS-30	Assemblers	Newbler version 2.3-PreRelease-June 30,2009, VELVET version 1.0.13, phrap version SPS - 4.24	Newbler version 2.3-PreRelease-June 30, 2009, VELVET version 1.0.13, phrap version SPS - 4.24
MIGS-32	Gene calling method	Prodigal 1.4, GenePRIMP	Prodigal 1.4, GenePRIMP
	INSDC ID	AEVP00000000	CP002736.1
	Genome Database release	December 10, 2010	August 13, 2012
	Genbank Date of Release	February 17, 2011	May 23, 2011
MIGS-13	GOLD ID NCBI project ID Source material identifier	Gi03933 46699 DSM 574^T^	Gc01783 50757 DSM 14880^T^
	Project relevance	Obtain insight into the phylogenetic and physiological diversity of *Desulfotomacum* species.	Obtain insight into the phylogenetic and physiological diversity of *Desulfotomacum* species, and hydrogenogenic CO conversion.

### Growth conditions and DNA isolation

*D. nigrificans* and *D. carboxydivorans* were grown anaerobically at 55^o^C in bicarbonate buffered medium with lactate and sulfate as substrates [6]. DNA of cell pellets was isolated using the standard DOE-JGI CTAB method recommended by the DOE Joint Genome Institute (JGI, Walnut Creek, CA, USA). Cells were resuspended in TE (10 mM tris; 1 mM EDTA, pH 8.0). Subsequently, cells were lysed using lysozyme and proteinase K, and DNA was extracted and purified using CTAB and phenol:chloroform:isoamylalcohol extractions. After precipitation in 2-propanol and washing in 70% ethanol, the DNA was resuspended in TE containing RNase. Following a quality and quantity check using agarose gel electrophoresis in the presence of ethidium bromide, and spectrophotometric measurement using a NanoDrop ND-1000 spectrophotometer (NanoDrop® Technologies, Wilmington, DE, USA).

### Genome sequencing and assembly

The genome of *D nigrificans* strain Delft 74 (DSM 574) was sequenced using a combination of Illumina and 454 sequencing platforms. All general aspects of library construction and sequencing can be found at the JGI website [24]. Pyrosequencing reads were assembled using the Newbler assembler (Roche). The initial Newbler assembly consisting of 75 contigs in two scaffolds was converted into a phrap [25] assembly by making fake reads from the consensus, to collect the read pairs in the 454 paired end library. Illumina GAii sequencing data (3,053.3 Mb) was assembled with Velvet [26] and the consensus sequences were shredded into 1.5 kb overlapped fake reads and assembled together with the 454 data. The 454 draft assembly was based on 127.9 Mb 454 draft data and all of the 454 paired end data. Newbler parameters are -consed -a 50 -l 350 -g -m -ml 21. The Phred/Phrap/Consed software package [25] was used for sequence assembly and quality assessment in the subsequent finishing process. After the shotgun stage, reads were assembled with parallel phrap (High Performance Software, LLC). Whenever possible mis-assemblies were corrected with gapResolution [24], Dupfinisher [27], or sequencing cloned bridging PCR fragments with subcloning. Some gaps between contigs were closed by editing in Consed, by PCR and by Bubble PCR primer walks (J.-F. Chang, unpublished). Some mis-assembly is still possible in the current assembly that consists in seven contigs and one scaffold. A total of 268 additional reactions and one shatter library were necessary to close gaps and to raise the quality of the final contigs. Illumina reads were also used to correct potential base errors and increase consensus quality using a software Polisher developed at JGI [28]. The error rate of the final genome sequence is less than 1 in 100,000. Together, the combination of the Illumina and 454 sequencing platforms provided 498.0× coverage of the genome. The final assembly contained 332,256 pyrosequence and 37,872,777 Illumina reads.

The same protocol applied to the *D. carboxydivorans* strain CO-1-SRB (DSM 14880) genome allowed to produce finished assembly without gaps. Illumina GAii sequencing data (334.0Mb) was assembled with Velvet 0.7.63 and the 454 draft assembly was based on 138.8 MB of sequence. A total of 290 additional reactions were necessary to close some gaps and to raise the quality of the final contigs. Illumina reads were also used to correct potential base errors and increase consensus quality using a software Polisher developed at JGI [28]. The error rate of the final genome sequence is less than 1 in 100,000. Together, the combination of the Illumina and 454 sequencing platforms provided 167.4× coverage of the genome. The final assembly contained 543,495 pyrosequence and 9,254,176 Illumina reads

### Genome annotation

Genes were identified using Prodigal [29] as part of the DOE-JGI genome annotation pipeline [30], followed by a round of manual curation using the JGI GenePRIMP pipeline [31]. The predicted CDSs were translated and used to search the National Center for Biotechnology Information (NCBI) nonredundant database, UniProt, TIGR-Fam, Pfam, PRIAM, KEGG, COG, and InterPro databases. Additional gene prediction analysis and functional annotation was performed within the Integrated Microbial Genomes - Expert Review (IMG-ER) platform [32].

## Genome properties

The genome of *D. nigrificans* and *D. carboxydivorans* consist of one chromosome of 3,052,787 and 2,892,255 nucleotides with a GC content of 46.28 and 46.63%, respectively (Table 4). Of the 3,112 genes in the genome of *D. nigrificans*, 98 are RNA genes of which 6 16S rRNA genes. A total of 2,340 genes of the 3,014 protein coding genes are assigned to COG functional categories. The distribution of these genes into COG functional categories is presented in Table 5. The distribution of the 2,174 COG assigned genes of *D. carboxydivorans* into COG functional categories is also presented in Table 5. Of the 2,844 predicted genes in the *D. carboxydivorans* genome, 2,747 are protein coding genes and 97 RNA genes, of which 8 are 16S rRNA genes. Both strains have sets of multiple 16S rRNA genes. Within the sets and among the sets most of the genes are 99.5-99.9% identical. Each strain has one differently deviating 16S rRNA gene, the difference probably originating from differential gene loss. In addition, 3.09% of the total genes of *D. carboxydivorans* are identified as pseudo genes. More genome statistics of *D. nigrificans* and *D. carboxydivorans* are displayed in Table 4.

**Table 4:** Genome statistics of DSM 574 (A) and DSM 14880 (B)

**Attribute**	A. Genome (total)		B. Genome (total)	
	Value	% of total	Value	% of total
Genome size (bp)	3,052,787	100	2,892,255	100.00
DNA coding region (bp)	2,595,629	85.02	2457154	84.96
DNA G+C content (bp)	1,412,511	46.28	1,348,537	46.63
Total genes	3,112	100	2,844	100
RNA genes	98	3.15	97	3.41
Protein-coding genes	3,014	96.85	2,747	96.59
Genes in paralog clusters	1,542	49.55	1,363	47.93
Genes assigned to COGs	2,340	75.19	2,174	76.44
Pseudo genes	137	4.40	88	3.09
Genes with signal peptides	582	18.70	504	17.72
Genes with transmembrane helices	721	23.17	647	22.75

**Table 5:** Number of DSM 574 and DSM 14880 genes associated with the general COG functional categories.

		DSM 574		DSM 14880	
Code	Description	Value	%age^a^	Value	%age^a^
J	Translation	153	5.97	152	3.39
A	RNA processing and modification	1	0.04	0	0.00
K	Transcription	153	5.97	139	5.85
L	Replication, recombination and repair	210	8.20	172	7.23
B	Chromatin structure and dynamics	1	0.04	1	0.04
D	Cell cycle control, mitosis and meiosis	45	1.76	45	1.89
Y	Nuclear structure	0	0.00	0	0.00
V	Defense mechanisms	22	0.86	22	0.93
T	Signal transduction mechanisms	171	6.71	148	6.22
M	Cell wall/membrane biogenesis	132	5.15	126	5.3
N	Cell motility	70	2.73	68	2.86
Z	Cytoskeleton	0	0.00	0	0.00
W	Extracellular structures	0	0.00	0	0.00
U	Intracellular trafficking and secretion	65	2.54	64	2.69
O	Posttranslational modification, protein turnover, chaperones	83	3.24	85	3.57
C	Energy production and conversion	217	8.47	211	8.87
G	Carbohydrate transport and metabolism	125	4.88	98	4.12
E	Amino acid transport and metabolism	224	8.74	216	9.08
F	Nucleotide transport and metabolism	62	2.42	60	2.52
H	Coenzyme transport and metabolism	134	5.23	133	5.59
I	Lipid transport and metabolism	40	1.56	36	1.51
P	Inorganic ion transport and metabolism	104	4.06	101	4.25
Q	Secondary metabolites biosynthesis, transport and catabolism	29	1.13	27	1.14
R	General function prediction only	261	10.19	250	10.51
S	Function unknown	241	9.41	224	9.42
-	Not in COGs	772	24.81	670	23.56

a) The total is based on the total number of protein coding genes in the annotated genome.

**Figure 4:**
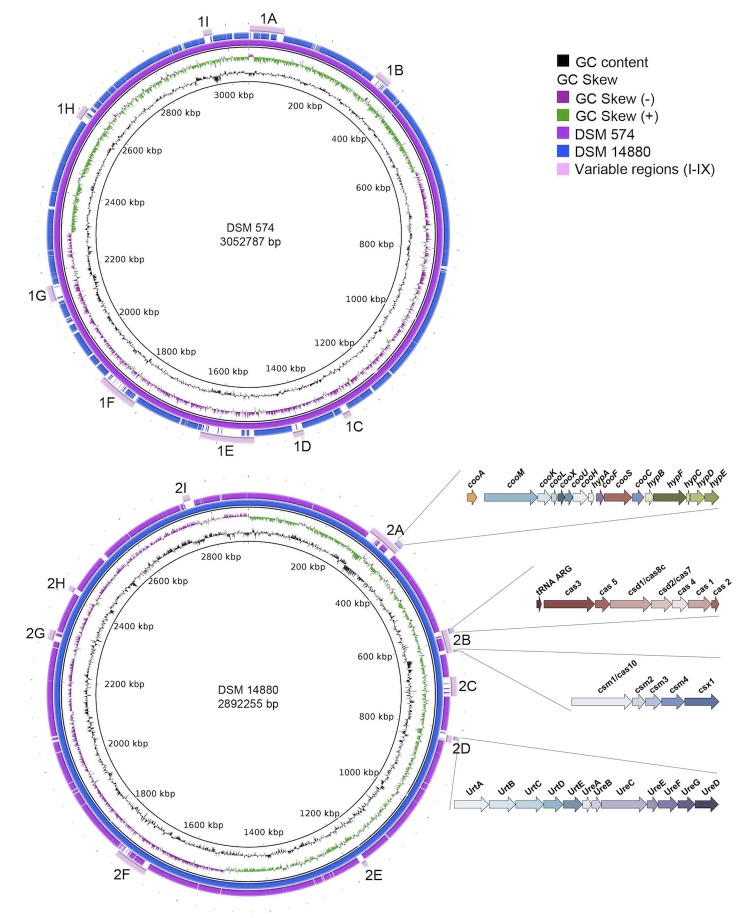
Graphical map of the DSM 574 (upper) and DSM 14880 (lower) chromosome. In both maps one genome was compared to the other. When genes were not similar or present in the other genome it resulted in gaps. The indicated variable regions with their function can also be found in Table 6 and the supplementary data S1.

**Table 6:** Description of genes present in the variable regions depicted in Figure 4.

**Variable region**	**Functions**
1A	Transposases, recombinases, transport proteins, isomerases, histidine kinase and threonine dehydrogenase
1B	Transposases, recombinases, resolvase and alcohol dehydrogenase
1C	Helicases, DNA-methylation, endonuclease and recombinase
1D	TRAP transporter, Threonine dehydrogenase, 2 keto-4-petnenoate hydratase, sugar kinase, aldolase, glycerol dehydrogenase and mannonate dehydratase
1E	Pilus assembly, proteases and hypothetical proteins dominate this variable region
1F	Protease, DNA methylase, RNA polymerase, recombinase, cytochrome c biogenesis, Fe^2+^ transport system and many hypothetical proteins
1G	Transposase, secretory protein secB, nucleotide sugar dehydrogenase, glycosyltransferase, sugar epimerase, O-antigen ligase and copper amine oxidase
1H	Pyruvate ferredoxin oxidareductase, transport proteins, sugar phosphate permease, threonine dehydrogenase, transporsase, DNA methylase and endonuclease
1I	Growth inhibitor protein, terminase, phage portal protein, secretory protein, recombinase and many hypothetical proteins
2A	Endonuclease, DNA methylase, transposase, ATP binding protein, ATPase, threonine kinase, pyridoxamine 5’phosphate oxidase, ferric reductase, many hypothetical proteins and the CODH-ECH complex
2B	CRISPR-Cas
2C	DNA-helicases, -methyltransferase, and -replication protein, restriction protein and many hypothetical proteins
2D	Urea metabolism
2E	Mainly transport proteins and agmatinase
2F	Alpha ribazole phosphatase, metal dependent phosphohydrolase, phenylacetate-CoA ligase, methyltransferase, amine oxidase, aldehyde dehydrogenase, transposase, phage tail component and many hypothetical proteins
2G	Pilus associated proteins
2H	Recombinase, integrase, AAA ATPase, restriction modification system, deoxyribonuclease
2I	Many transferase proteins

## Insights into the genomes

### Incomplete oxidation of organic compounds

*D. nigrificans* and *D. carboxydivorans* oxidize organic substrates such as lactate, pyruvate, ethanol and sugars incompletely to acetate. Both genomes have gene copies that are predicted to encode L-lactate dehydrogenases (DesniDRAFT_1264, 2906; Desca_0533) and D-lactate dehydrogenase (DesniDRAFT_0054, 1145, 1691; Desca_0863, 2222), which are involved in the oxidation of lactate to pyruvate. For incomplete oxidation of pyruvate to acetate via acetyl-CoA *D. nigrificans* and *D. carboxydivorans* have genes encoding a putative pyruvate dehydrogenase (DesniDRAFT_1250, 2504, 1245 and Desca_0770, 0146, 0775, respectively) and subsequently an acetyl-CoA synthetase (DesniDRAFT_2242 and Desca_0484, respectively). Although the two strains cannot grow with succinate, fumarate and malate as electron donors, genes to metabolize these compounds are present in both genomes. *D. nigrificans* and *D. carboxydivorans* have genes putatively coding for a fumarate reductase (DesniDRAFT_0617-15 and Desca_1387-89), fumarate hydratase (DesniDRAFT_0612-13 and Desca_1391-92), malate dehydrogenase (DesniDRAFT_0618 and Desca_1386), and a pyruvate carboxylase (DesniDRAFT_1477-78 and Desca_2116-17) that might be involved in the oxidation of succinate, fumarate and malate to pyruvate. For growth on ethanol, both genomes contain alcohol dehydrogenases (DesniDRAFT_0051, 0320, 0326, 0367, 1219, 2126, 2174, 2779; Desca_0375, 0418, 1671, 1913, 1943, 2553, 2558) and acetaldehyde dehydrogenases (DesniDRAFT_0038; Desca_1928).

For sulfate reducers to oxidize acetate to CO_2_, either the complete tricarboxylic acid (TCA) cycle or acetyl-CoA pathway has to be present [33]. Since *D. nigrificans* and *D. carboxydivorans* cannot grow with acetate, it was expected neither strain would possess a complete TCA cycle; which was verified by a lack of the putative genes that code for ATP-dependent citrate synthase, aconitase, and isocitrate dehydrogenase. All genes coding for the acetyl-CoA pathway are present in both genomes, except for the genes encoding the acetyl-CoA synthase subunit and the FeS-protein large and small subunit. Probably the gene coding for the acetyl-CoA synthetase is also involved in the acetyl-CoA production from acetate and coenzyme A.

### Sugar metabolism

*D. nigrificans* and *D. carboxydivorans* are able to utilize glucose and fructose as electron donors in the presence of sulfate. Additionally, both species are able to ferment fructose, although fermentation of glucose is only reported for *D. carboxydivorans* [5,6]. The capability of utilizing sugars for growth is unusual among *Desulfotomaculum* species. The other *Desulfotomaculum* species that belong to cluster I, sub group a, *D. ruminis*, *D. aeronauticum*, *D. putei* and *D. hydrothermale* (with the exception of “*D. reducens”*), are not able to grow with glucose or fructose [34-36]. Glucose metabolism in *D. nigrificans* was studied before [4]. Akagi and Jackson showed that the majority of the glucose was degraded by the Embden-Meyerhof-Parnas pathway and in several instances the glucose followed the Entner-Doudoroff pathway [4]. The Embden-Meyerhof-Parnas pathway and the pentose phosphate pathway are predicted to be complete in the genome of *D. nigrificans* and *D. carboxydivorans*. However, genes coding for the 6-phosphogluconate dehydratase and the 2-keto-3-deoxy-6-phosphogluconate aldolase, the two characteristic enzymes of the Entner-Doudoroff pathway, were not found in the genome of *D. nigrificans* and *D. carboxydivorans.* A phosphotransferase system (PTS) for glucose-specific transport was not found in either genome. Such a system is present in the genome of the glucose-utilizer *D. reducens* (Dred_0332). Genes coding for the fructose-specific PTS are present in an operon structure in *D. nigrificans* (DesniDRAFT_2286 and 2291) and *D. carboxydivorans* (Desca_2698 and 2703). This system is likely involved in fructose uptake and its subsequent phosphorylation to fructose-1-phosphate. The fructose-1-phosphate thus formed can be further phosphorylated by 1-phosphofructokinase to fructose-1,6-bisphosphate (DesniDRAFT_2290 and Desca_2702).

Unlike *D. nigrificans* and *D. carboxydivorans*, *D. ruminis* and *D. kuznetsovii* are not able to grow with glucose or fructose. However, they have the genes that code for all the enzymes involved in the Embden-Meyerhof-Parnas pathway present in their genome. What is missing in their genome is the PTS for fructose-specific transport. This suggests that the absence of this PTS system prevents the use of fructose for growth.

### Growth on one-carbon substrates

*D. nigrificans* and *D. carboxydivorans* can grow with formate plus sulfate in the presence of yeast extract and acetate as a carbon source. Since the genomes lack a complete acetyl-CoA pathway, *D. nigrificans* and *D. carboxydivorans* are not able to produce acetyl-CoA from formate and need an additional carbon source. The two genomes have similar genes that putatively code for three formate dehydrogenases (FDHs). The first FDH consists of an alpha subunit (DesniDRAFT_0989, Desca_1018), which is located next to a hydrogenase (DesniDRAFT_0990, Desca_1017) and a flavoprotein (DesniDRAFT_0988 and Desca_1019). The flavoprotein has one predicted transmembrane helix. Therefore, these genes might code for one intracellular membrane associated FDH. The second FDH gene cluster (DesniDRAFT_1389-1392, Desca_2053-2055) putatively codes for a confurcating cytoplasmic FDH. The third is predicted to code for an extracellular FDH (DesniDRAFT_1396-1397, Desca_2059-2060) associated with the membrane by a proposed 10 transmembrane helixes containing protein (DesniDRAFT_1395, Desca_2058). BLAST results and orthologous BLAST analysis [37] indicate that this transmembrane helix protein is orthologous to cytochrome b. Therefore, electron transport from this FDH might go through cytochrome b.

**Figure 5:**
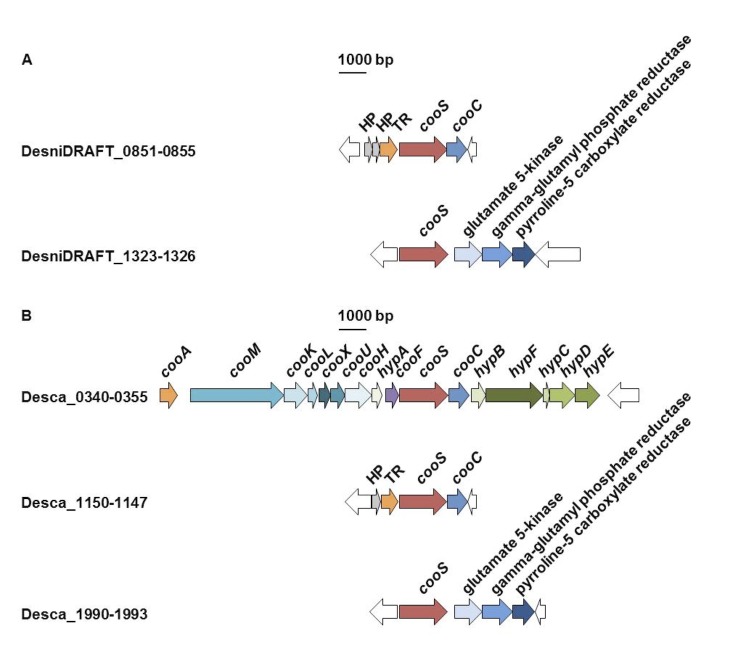
Organization of *cooS* and neighboring genes in DSM 574 (A) and DSM 14880 (B). Abbreviations: HP, hypothetical protein; TR, transcriptional regulator.

*D. nigrificans* and *D. carboxydivorans* are able to grow with CO in the presence of yeast extract. However, *D. nigrificans* grows with up to 20% of CO coupled to sulfate reduction, while *D. carboxydivorans* can grow with 100% CO with and without sulfate. These physiological differences should also be visible in the genome for the genes involved with carbon monoxide dehydrogenase (CODH). Figure 5 shows the organization of the CODH catalytic subunit (*cooS*) and neighboring genes in *D. nigrificans* and *D. carboxydivorans*. *D. nigrificans* has two *cooS* genes in the genome (DesniDRAFT_0854 and 1323) while *D. carboxydivorans* has three (Desca_0349, 1148, 1990). The organization of the *cooS* and neighboring genes in *D. nigrificans* is similar to that of two of the *cooS* and neighboring genes in *D. carboxydivorans*. However, one *cooS* gene cluster in the *D. carboxydivorans* genome cannot be found in the genome of *D. nigrificans*. The genes in this cluster are similar to genes described to be involved in the H_2_ production from CO oxidation [38-41]. *Carboxydothermus hydrogenoformans* was the first bacterium described to have multiple *cooS* genes, one of which is united in a cluster with hydrogenase genes [40]. The hydrogenase module of this gene cluster represents a membrane-bound energy-converting hydrogenase (ECH) capable of energizing the membrane by proton translocation. Among sequenced *Desulfotomaculum* species, only *D. carboxydivorans, D. acetoxidans*, and *D. ruminis* possess putative genes coding for ECHs. However, in the latter two genomes, ECH encoding genes do not cluster with *cooS* genes. Earlier analysis showed that clustering of cooS genes and ECH genes is a characteristic feature of hydrogenogenic carboxydotrophs [42]. The presence of the putative ECH-cooS gene cluster in *D. carboxydivorans* explains its ability to grow hydrogenogenically on CO.

In *D. nigrificans* there are no CODH involved genes in close proximity of the *cooS* genes, apart from one *cooC* gene (DesniDRAFT_0855). Apparently, this is sufficient for *D. nigrificans* to grow with 20% of CO coupled to sulfate reduction. However, *D. ruminis*, another *Desulfotomaculum* species in cluster 1a (Figure 1) of which the genome was recently described [43], also has the *cooS* gene (Desru_0859) downstream of a transcriptional regulator (Desru_0858) and upstream of the *cooC* gene (Desru_0860) but that bacterium is not able to grow on CO and sulfate. The reason for this is not yet clear.

A cluster of nitrogenase genes (Dtox_1023 to 1030) has been described in the genome of *Desulfotomaculum acetoxidans* [44]. In the genomes of *D. nigrificans* and *D. carboxydivorans* very similar gene clusters occur (DesniDRAFT_0869-0858 and Desca_1134-1144). Notably, in both cases there are *cooS* genes in the vicinity (DesniDRAFT_0854 and Desca_1148). They are located on another DNA strand and are convergently directed. Since the low-potential carbon monoxide seems to be a good electron donor for nitrogen fixation, this proximity might be more than mere coincidence. This would suggest that small amounts of CO could be oxidized by *D. nigrificans* in the absence of sulfate. *D. ruminis* also has a similar gene cluster (Desru_3454-3445). However, in contrast to the genomes of *D. nigrificans* and *D. carboxydivorans* no cooS gene is nearby in the genome of *D. ruminis*.

Methyltransferase genes as present in *D. kuznetsovii* that might point to possible growth with methanol or methylated amines were not found in the genomes of *D. nigrificans* and *D. carboxydivorans*. These two strains accordingly, do not grow with methanol. Growth on methylated amines were never tested, but the genome suggests there is no growth possible with these compounds.

### Hydrogen metabolism

*D. nigrificans* and *D. carboxydivorans* have a similar hydrogenase composition that is dominated by [FeFe] hydrogenases, as observed in other *Desulfotomaculum* spp. Each of the two bacteria has 9 [FeFe] hydrogenases, divided in the following groups: Three copies of trimeric bifurcating hydrogenases (DesniDRAFT_0775-0777, DesniDRAFT_0770-0772 and DesniDRAFT_1331-1333; Desca_1224-1226, Desca_1230-1232 and Desca_1996-1998); two copies of a monomeric hydrogenase (DesniDRAFT_0646 and DesniDRAFT_0308; Desca_1356 and Desca_1680); one HsfB-type hydrogenase encoding a PAS-sensing domain that is likely involved in sensing and regulation (DesniDRAFT_0986 and Desca_1021); one hydrogenase that is part of a 5-gene operon also encoding one membrane protein and two flavin-dependent oxidoreductases (DesniDRAFT_1073-1077 and Desca_0931-0935); and finally two copies of a membrane-associated hydrogenase (DesniDRAFT_1068-1070 and DesniDRAFT_2001-2003; Desca_0940-0938 and Desca_2453-2455). The catalytic subunit (DesniDRAFT_1068, 2001 and Desca_0940, 2453) of this hydrogenase contains a tat signal motif, which suggests that the hydrogenase complex is positioned extracellular. Moreover, the membrane associated subunit is a 10 transmembrane helix containing protein that is orthologous to cytochrome b. This is similar to the extracellular FDH.

The high number of hydrogenases in the genomes of the two bacteria indicate a high metabolic flexibility. This is important for changing growth strategies, from, for example, sulfate respiration to syntrophic growth. A syntrophic co-culture of *D. nigrificans* and *Methanobacterium thermoautotrophicum* on lactate and ethanol was described [5]. Syntrophic consortia are able to grow from very small free energy changes due to their ability to overcome thermodynamically difficult reactions. Reverse electron transfer is an essential part of this. The genes coding for the bifurcating hydrogenases and the confurcating formate dehydrogenase in the *D. nigrificans* genome are therefore likely candidates to be involved in syntrophic growth on lactate and ethanol.

A membrane-associated ECH is present only in *D. carboxydivorans*, as mentioned above, and no other [NiFe] hydrogenases are present. Other membrane associated complexes found in the genome of *D. nigrificans* and *D. carboxydivorans* are complex I (DesniDRAFT_0902-0892 and Desca_1110-1120) and a H^+^-pumping membrane-bound pyrophosphatase (DesniDRAFT_2060 and Desca_2506).

### Electron acceptor metabolism

The genes for the assimilatory sulfate reduction are organized in an identical way in *D. nigrificans* and *D. carboxydivorans*. ATP-sulfurylase (DesniDRAFT_1837, Desca_2237) is followed by adenosine-5´-phosphosulfate (APS) reductase (DesniDRAFT_1836-1835, Desca_2378-2377), and the QmoAB complex (DesniDRAFT_1834-1833, Desca_ 2376-2375). A qmoC gene is absent but seems to be substituted by heterodisulfide reductases (Hdr) CB (DesniDRAFT _1838-1839, Desca_ 2381-2380). This organization is also found in *D. ruminis* and *D. reducens*. The position of the HdrCB is switched to the other side in *D. acetoxidans*, *D. gibsoniae*, *D. alcoholicoviorans*, *Desulfurispora thermophila*, and *Desulfarculus baarsii* (which owns a Gram-positive aprBA [45]). In contrast to these organisms, *D. kuznetsovii*, *Ammonifex degensii*, *Desulfovirgula thermocuniculi*, and Gram-negative sulfate-reducing bacteria which posses a Gram-positive aprBA [45] like *Desulfomonile tiedjei* and *Syntrophobacter fumaroxidans* have a complete qmoABC complex (for *D. kuznetsovii*: Desku_ 1075, Desku_1076, Desku_1078).

The genes for the dissimilatory sulfite reductase found and their organization are identical to all other six *Desulfotomaculum* genomes published so far and most other Gram-positive sulfate-reducing bacteria. The dsrAB genes (DesniDRAFT_2256-2255, Desca_2666-2665) are linked to a dsrD gene (DesniDRAFT_2254, Desca_2664). Both organisms also contain a truncated DsrMK complex [46](DesniDRAFT_2267-2268, Desca_2678-2679) which is linked to a dsrC gene (DesniDRAFT_2266, Desca_2677) as it was found in *D. ruminis* [43]. This truncated DsrMK is generally found in Gram-positive sulfate-reducing bacteria and not restricted to members of the genus *Desulfotomaculum*.

*D. nigrificans* and *D. carboxydivorans* lack nitrate reduction genes for reduction of nitrate to N_2_. Nitrate reductase, nitric-oxide forming nitrite reductase, nitric-oxide reductase and nitrous-oxide reductase are all absent in both genomes. However, a nitrite/sulphite reductase (DesniDRAFT_1001, 2506; Desca_0162, 1181) and an ammonia forming nitrite reductase (DesniDRAFT_0204; Desca_2313) are present in the genome of *D. nigrificans* and *D. carboxydivorans.* No taurine degradation pathway was detected in the genome of either strain, but it was described for the closely related *D. ruminis* [43].

### Fumarate reductases

**Figure 6:**
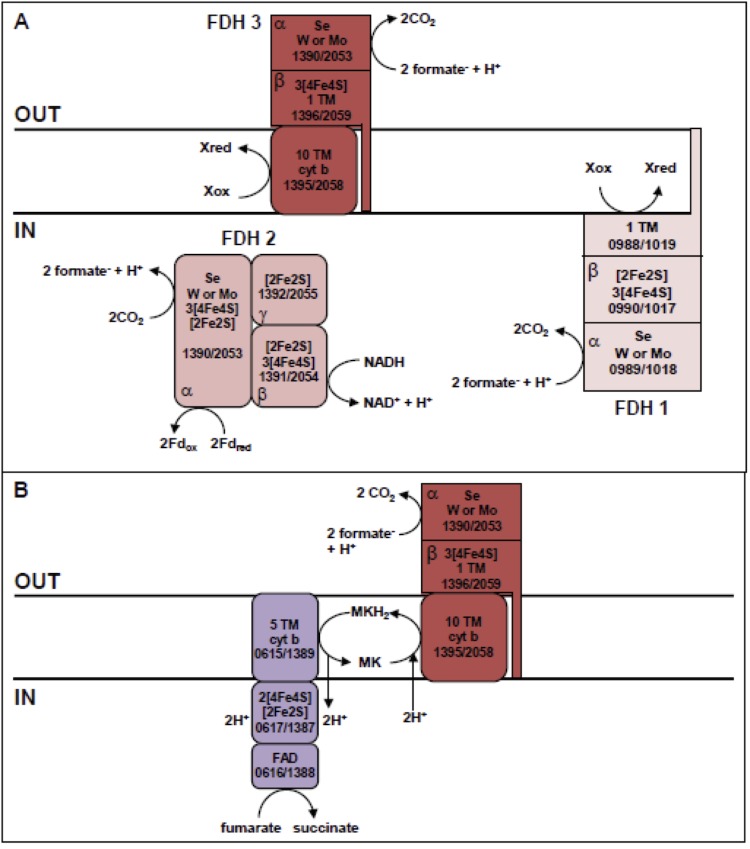
Schematic representation of putative formate dehydrogenases in the genome of DSM 574 and DSM 14880 (A). Including the hypothesized electron interaction of the putative extracellular membrane bound formate dehydrogenase with the putative fumarate reductase (B). The electron acceptor fumarate is reduced to succinate by using formate as an electron donor. Gene locus tag numbers and α-, β-, and γ-subunits are depicted. Moreover, predicted iron-sulfur clusters and other metal-binding sites are indicated.

Using fumarate as an electron acceptor for growth of *D. nigrificans* and *D. carboxydivorans* has not been tested yet. However, a fumarate reductase is present in the genomes of the two bacteria. The three genes encode for a FAD containing catalytic subunit (DesniDRAFT_0617; Desca_1387), an iron sulfur containing subunit (DesniDRAFT_0616; Desca_1388), and a membrane associated cytochrome b (DesniDRAFT_0615 and Desca_1389). This cytochrome b protein might perform an electron interaction with the cytochrome b of the extracellular FDH (Figure 6, panel B). This interaction could occur as described in *Wolinella succinogenes,* where fumarate can be used as an electron acceptor for growth on formate [47].

## Comparative genomics

### Distinct genes in *Desulfotomaculum carboxydivorans* and *D. nigrificans*

**Figure 7:**
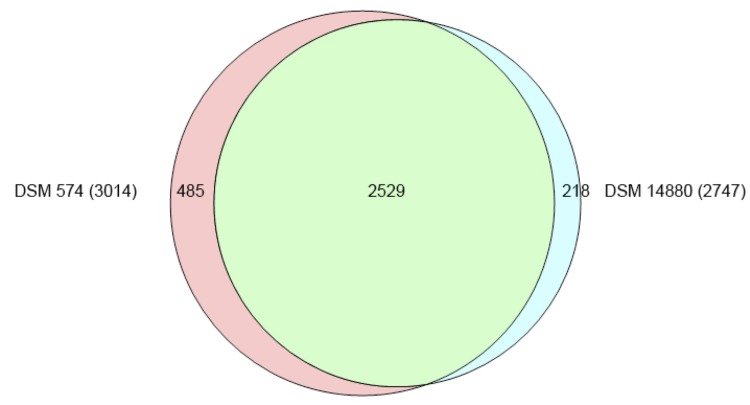
Venn diagram showing a comparison of the protein coding genes of DSM 574 and DSM 14880. The number of overlapping protein coding genes is given inside the areas of the circles and the total number of derived protein sequences used for each strain is shown in parentheses. The figure was created using the program Venn diagram plotter available from the Pacific Northwest National Laboratory Software Distribution Center [48].

To reveal genomic differences between these two very closely related species, a bidirectional BLAST of the protein coding genes was performed. BLAST analyses were performed using standard settings and best hits were filtered for 70% sequence coverage and 40% identity (supplementary data S1). A total of 2,529 homologous genes were found (Figure 7). The distinct genes were screened for operon structure and function, revealing genes involved in CRISPR, urea metabolism and hydrogenogenic CO metabolism in *D. carboxydivorans*.

CRISPR genes in *D. carboxydivorans* were found to have low sequence coverage and or identity with genes in the *D. nigrificans* genome (Figure 3). These genes involved two CRISPR-Cas systems, which we classified as a I-C subtype (Desca_0534-0540) and a III-A subtype (Desca_0572-0576). *D. nigrificans* has one CRISPR-Cas system subtype, I-A (DesniDRAFT_2444-2452), which is also present in *D. carboxydivorans* (Desca_0726-0734). The presence of multiple CRISPR-Cas systems and the occurrence of the different subtypes in one strain has been described previously [49,50] and shows that the co-occurrence of subtype I-A with I-C and III-A is a common feature. However, it also shows that *D. carboxydivorans* is part of the 2% of bacteria that have a III-A subtype without a III-B subtype.

The genome of *D. carboxydivorans* also contains genes coding for a urease (Desca_0743-0749) and urea transport (Desca_0738-0742) (Figure 3). Urease catalyzes the reaction of urea to CO_2_ and ammonia. Urea is very common in the environment and is a nitrogen source for many bacteria [51]. The genome of *D. nigrificans* lacks the genes coding for an urease, which indicates that *D. nigrificans* is relatively more restricted regarding its nitrogen source. Other interesting genes that are present in the *D. carboxydivorans* genome and not in the *D. nigrificans* genome are genes involved in the carbon monoxide dehydrogenase (CODH) and hydrogenase as described above.

### Taxonomic conclusions

The overall similarity of the genome sequences of the type strains of *D. nigrificans* and *D. carboxydivorans* was estimated by using the Genome-To-Genome Distance Calculator (GGDC) as described previously [52]. This program calculates DNA-DNA similarity values by comparing the genomes to obtain high-scoring segment pairs (HSPs) and inferring distances from a set of three formulas (1, HSP length/total length; 2, identities/HSP length; 3, identities/total length). According to the GGDC the average estimated DNA-DNA similarity value between the two type strains is 86.5 ± 5.5% and thus clearly above 70%, which is the widely accepted threshold value for assigning strains to the same species [53]. The high similarity of the genome sequences of both type strains was further supported by the average nucleotide identity of shared genes (ANI), which proved to be above 99%. This ANI value is much higher than the 95 to 96% value shown to correspond to the 70% DNA-DNA hybridization level [54]. Moreover, the two strains have almost identical 16S rRNA gene sequences (>99%) and a high number of shared genes (Figure 7). It should be mentioned that the previously reported and deposited rRNA gene sequence of *D. nigrificans* DSM 574 contained a lot ambiguities and some missing nucleotides, which are counted as mismatches by BLAST. Therefore, we reanalyzed the rRNA gene sequences of *D. nigrificans* deposited in the NCIMB culture collections and confirmed the identity of the rRNA gene sequence found in the genome of DSM 574. We propose that the species should be united under one name. According to the rules of priority as given by the Bacteriological Code [55] the name *D. nigrificans* should be used for the unified taxon, with *D. carboxydivorans* as a later heterotypic synonym.

## Emended description of *Desulfotomaculum nigrificans* (Werkman and Weaver 1927) Campbell and Postgate 1965

The description is as given by Campbell and Postgate [1] and Parshina et al. [6] with the following modifications.

The cells are Gram-positive, rod-shaped with rounded ends, 0.3-1.5 x 2-15 µm, single or sometimes paired. Motility with tumbling or twisting movements conferred by peritrichous flagella. Terminal or subterminal oval endospores that are slightly swelling the cells. Thermophilic and neutrophilic with an temperature optimum of 55°C. NaCl is not required for growth. The following substrates are utilized, coupled to the reduction of sulfate to sulfide: DL-lactate, pyruvate, ethanol, L-alanine, D-fructose, D-glucose. Acetate and methanol are not utilized. Substrates are incompletely oxidized to acetate. In the presence of 0.5 g/l yeast extract, lithoheterotrophic growth is possible, such as growth on H_2_ and CO_2_ with sulfate or growth on 20% CO with sulfate for *D. nigrificans* strain Delft 74 and growth on 100% CO with or without sulfate for strain CO-1-SRB. Suitable electron acceptors with lactate as substrate are sulfate, sulfite and thiosulfate, but not elemental sulfur or nitrate. Fermentation of pyruvate and fructose; strain CO-1-SRB is also able to ferment DL-lactate, glucose and CO. The prevalent respiratory lipoquinone is MK7 with only small amounts of MK6. The dominating cytochromes are of type *b*. Major cellular fatty acids are 16:0, iso 15:0, iso 17:0, anteiso 15:0, 18:0 and iso 16:0. The DNA G+C content is around 46 mol%. The type strain is Delft 74 (=NCIMB 8395 = DSM 574 = ATCC 19998 = NBRC 13698).

## References

[1] Campbell LL Postgate JR Classification of the spore-forming sulfate-reducing bacteria. Bacteriol Rev 1965; 29:359-363 5826606 10.1128/br.29.3.359-363.1965 PMC441283

[2] Werkman CH Weaver HJ Studies in the bacteriology of sulphur stinker spoilage of canned sweet corn. State Coll. J. Sci. 1927; 2:57-67

[3] Starkey RL A study of spore formation and other morphological characteristics of *Vibrio desulfuricans.* Arch Mikrobiol 1938; 9:268-404 10.1007/BF00407364

[4] Akagi JM Jackson G Degradation of glucose by proliferating cells of *Desulfotomaculum nigrificans.* Appl Microbiol 1967; 15:1427-1430 16349758 10.1128/am.15.6.1427-1430.1967 PMC547220

[5] Klemps R Cypionka H Widdel F Norbert P Growth with hydrogen, and further physiological characteristics of *Desulfotomaculum* species. Arch Microbiol 1985; 143:203-208 10.1007/BF00411048

[6] Parshina SN Sipma J Nakashimada Y Henstra AM Smidt H Lysenko AM Lens PN Lettinga G Stams AJ *Desulfotomaculum carboxydivorans* sp. nov., a novel sulfate-reducing bacterium capable of growth at 100% CO. Int J Syst Evol Microbiol 2005; 55:2159-2165 10.1099/ijs.0.63780-0 16166725

[7] Postgate JR Sulfate-Free Growth of *Clostridium nigrificans.* J Bacteriol 1963; 85:1450-1451 14047249 10.1128/jb.85.6.1450-1451.1963 PMC278360

[8] Krishnamurthi S Spring S Anil Kumar P Mayilraj S Klenk HP Suresh K *Desulfotomaculum defluvii* sp. nov., a sulfate-reducing bacterium isolated from the subsurface environment of a landfill. Int J Syst Evol Microbiol 2012 23159750 10.1099/ijs.0.047662-0

[9] Collins M Weddel F Respiratory quinones of sulphate-reducing and sulphur-reducing bacteria: a systematic investigation. Syst Appl Microbiol 1986; 8:8-18 10.1016/S0723-2020(86)80141-2

[10] Field D Garrity G Gray T Morrison N Selengut J Sterk P Tatusova T Thomson N Allen MJ Angiuoli SV The minimum information about a genome sequence (MIGS) specification. Nat Biotechnol 2008; 26:541-547 10.1038/nbt1360 18464787 PMC2409278

[11] Woese CR Kandler O Wheelis ML Towards a natural system of organisms: proposal for the domains Archaea, Bacteria, and Eucarya. Proc Natl Acad Sci USA 1990; 87:4576-4579 10.1073/pnas.87.12.4576 2112744 PMC54159

[12] Gibbons NE Murray RGE Proposals Concerning the Higher Taxa of Bacteria. Int J Syst Bacteriol 1978; 28:1-6 10.1099/00207713-28-1-1

[13] Garrity GM, Holt JG. The Road Map to the Manual. In: Garrity GM, Boone DR, Castenholz RW (eds), Bergey's Manual of Systematic Bacteriology, Second Edition, Volume 1, Springer, New York, 2001, p. 119-169.

[14] Murray RGE. The Higher Taxa, or, a Place for Everything...? In: Holt JG (ed), Bergey's Manual of Systematic Bacteriology, First Edition, Volume 1, The Williams and Wilkins Co., Baltimore, 1984, p. 31-34.

[15] List Editor List of new names and new combinations previously effectively, but not validly, published. List no. 132. Int J Syst Evol Microbiol 2010; 60:469-472 10.1099/ijs.0.022855-0 20458120

[16] Rainey FA. Class II. *Clostridia* class nov. In: De Vos P, Garrity G, Jones D, Krieg NR, Ludwig W, Rainey FA, Schleifer KH, Whitman WB (eds), Bergey's Manual of Systematic Bacteriology, Second Edition, Volume 3, Springer-Verlag, New York, 2009, p. 736.

[17] Skerman VBD McGowan V Sneath PHA Approved Lists of Bacterial Names. Int J Syst Bacteriol 1980; 30:225-420 10.1099/00207713-30-1-225 20806452

[18] Prévot AR. In: Hauderoy P, Ehringer G, Guillot G, Magrou. J., Prévot AR, Rosset D, Urbain A (eds), Dictionnaire des Bactéries Pathogènes, Second Edition, Masson et Cie, Paris, 1953, p. 1-692.

[19] Rogosa M *Peptococcaceae*, a new family to include the Gram-positive, anaerobic cocci of the genera *Peptococcus*, *Peptostreptococcus* and *Ruminococcus*. Int J Syst Bacteriol 1971; 21:234-237 10.1099/00207713-21-3-234

[20] Campbell LL Postgate JR Classification of the spore-forming sulfate-reducing bacteria. Bacteriol Rev 1965; 29:359-363 5826606 10.1128/br.29.3.359-363.1965 PMC441283

[21] Campbell LL. Genus IV. *Desulfotomaculum* Campbell and Postgate 1965, 361. In: Buchanan RE, Gibbons NE (eds), Bergey's Manual of Determinative Bacteriology, Eighth Edition, The Williams and Wilkins Co., Baltimore, 1974, p. 572-573.

[22] Ashburner M Ball CA Blake JA Botstein D Butler H Cherry JM Davis AP Dolinski K Dwight SS Eppig JT Gene ontology: tool for the unification of biology. The Gene Ontology Consortium. Nat Genet 2000; 25:25-29 10.1038/75556 10802651 PMC3037419

[23] Pagani I Liolios K Jansson J Chen IM Smirnova T Nosrat B Markowitz VM Kyrpides NC The Genomes OnLine Database (GOLD) v.4: status of genomic and metagenomic projects and their associated metadata. Nucleic Acids Res 2012; 40:D571-D579 10.1093/nar/gkr1100 22135293 PMC3245063

[24] JGI website. http://www.jgi.doe.gov/

[25] The Phred/Phrap/Consed software package. http://www.phrap.com

[26] Zerbino DR Birney E Velvet: algorithms for de novo short read assembly using de Bruijn graphs. Genome Res 2008; 18:821-829 10.1101/gr.074492.107 18349386 PMC2336801

[27] Han C, Chain P. Finishing repeat regions automatically with Dupfinisher. In: H.R. A, H. V, editors2006 June 26-29, 2006. CSREA Press. p 141-6.

[28] Lapidus A, LaButti K, Foster B, Lowry S, Trong S, Goltsman E. POLISHER: An effective tool for using ultra short reads in microbial genome assembly and finishing. Marco Island, FL: AGBT; 2008.

[29] Hyatt D Chen GL Locascio PF Land ML Larimer FW Hauser LJ Prodigal: prokaryotic gene recognition and translation initiation site identification. BMC Bioinformatics 2010; 11:119 10.1186/1471-2105-11-119 20211023 PMC2848648

[30] Mavromatis K Ivanova NN Chen IM Szeto E Markowitz VM Kyrpides NC The DOE-JGI Standard operating procedure for the annotations of microbial genomes. Stand Genomic Sci 2009; 1:63-67 10.4056/sigs.632 21304638 PMC3035208

[31] Pati A Ivanova NN Mikhailova N Ovchinnikova G Hooper SD Lykidis A Kyrpides NC GenePRIMP: a gene prediction improvement pipeline for prokaryotic genomes. Nat Methods 2010; 7:455-457 10.1038/nmeth.1457 20436475

[32] Markowitz VM Mavromatis K Ivanova NN Chen IM Chu K Kyrpides NC IMG ER: a system for microbial genome annotation expert review and curation. Bioinformatics 2009; 25:2271-2278 10.1093/bioinformatics/btp393 19561336

[33] Goevert D Conrad R Carbon isotope fractionation by sulfate-reducing bacteria using different pathways for the oxidation of acetate. Environ Sci Technol 2008; 42:7813-7817 10.1021/es800308z 19031865

[34] Hagenauer A Hippe H Rainey FA *Desulfotomaculum aeronauticum* sp. nov., a Sporeforming, Thiosulfate-Reducing Bacterium from Corroded Aluminium Alloy in an Aircraft. Syst Appl Microbiol 1997; 20:65-71 10.1016/S0723-2020(97)80049-5

[35] Haouari O Fardeau ML Cayol JL Casiot C Elbaz-Poulichet F Hamdi M Joseph M Ollivier B *Desulfotomaculum hydrothermale* sp. nov., a thermophilic sulfate-reducing bacterium isolated from a terrestrial Tunisian hot spring. Int J Syst Evol Microbiol 2008; 58:2529-2535 10.1099/ijs.0.65339-0 18984688

[36] Liu Y Karnauchow TM Jarrell KF Balkwill DL Drake GR Ringelberg D Clarno R Boone DR Description of two new thermophilic *Desulfotomaculum* spp., *Desulfotomaculum putei* sp. nov., from a deep terrestrial subsurface, and *Desulfotomaculum luciae* sp. nov., from a hot spring. Int J Syst Bacteriol 1997; 47:615-621 10.1099/00207713-47-3-615

[37] Zhou Y, Landweber LF. BLASTO: a tool for searching orthologous groups. Nucleic Acids Res 2007;35(Web Server issue):W678-82. 10.1093/nar/gkm278 PMC1933156 17483516

[38] Hedderich R Energy-converting [NiFe] hydrogenases from archaea and extremophiles: ancestors of complex I. J Bioenerg Biomembr 2004; 36:65-75 10.1023/B:JOBB.0000019599.43969.33 15168611

[39] Meuer J Bartoschek S Koch J Kunkel A Hedderich R Purification and catalytic properties of Ech hydrogenase from *Methanosarcina barkeri.* Eur J Biochem 1999; 265:325-335 10.1046/j.1432-1327.1999.00738.x 10491189

[40] Wu M Ren Q Durkin AS Daugherty SC Brinkac LM Dodson RJ Madupu R Sullivan SA Kolonay JF Haft DH Life in hot carbon monoxide: the complete genome sequence of *Carboxydothermus hydrogenoformans* Z-2901. PLoS Genet 2005; 1:e65 10.1371/journal.pgen.0010065 16311624 PMC1287953

[41] Fox JD He Y Shelver D Roberts GP Ludden PW Characterization of the region encoding the CO-induced hydrogenase of *Rhodospirillum rubrum.* J Bacteriol 1996; 178:6200-6208 8892819 10.1128/jb.178.21.6200-6208.1996 PMC178490

[42] Sokolova TG Henstra AM Sipma J Parshina SN Stams AJ Lebedinsky AV Diversity and ecophysiological features of thermophilic carboxydotrophic anaerobes. FEMS Microbiol Ecol 2009; 68:131-141 10.1111/j.1574-6941.2009.00663.x 19573196

[43] Spring S Visser M Lu M Copeland A Lapidus A Lucas S Cheng JF Han C Tapia R Goodwin LA Complete genome sequence of the sulfate-reducing firmicute *Desulfotomaculum ruminis* type strain (DL(T)). Stand Genomic Sci 2012; 7:294-309 10.4056/sigs.3226659 23408247 PMC3569383

[44] Spring S Lapidus A Schroder M Gleim D Sims D Meincke L Glavina Del Rio T Tice H Copeland A Cheng JF Complete genome sequence of *Desulfotomaculum acetoxidans* type strain (5575). Stand Genomic Sci 2009; 1:242-253 10.4056/sigs.39508 21304664 PMC3035247

[45] Meyer B Kuever J Phylogeny of the alpha and beta subunits of the dissimilatory adenosine-5'-phosphosulfate (APS) reductase from sulfate-reducing prokaryotes--origin and evolution of the dissimilatory sulfate-reduction pathway. Microbiology 2007; 153:2026-2044 10.1099/mic.0.2006/003152-0 17600048

[46] Junier P Junier T Podell S Sims DR Detter JC Lykidis A Han CS Wigginton NS Gaasterland T Bernier-Latmani R The genome of the Gram-positive metal- and sulfate-reducing bacterium *Desulfotomaculum reducens* strain MI-1. Environ Microbiol 2010; 12:2738-2754 20482743 10.1111/j.1462-2920.2010.02242.x PMC5662442

[47] Kroger A Biel S Simon J Gross R Unden G Lancaster CR Fumarate respiration of *Wolinella succinogenes*: enzymology, energetics and coupling mechanism. Biochim Biophys Acta 2002; 1553:23-38 10.1016/S0005-2728(01)00234-1 11803015

[48] Venn diagram plotter available from the Pacific Northwest National Laboratory Software Distribution Center: http://omics.pnl.gov

[49] Staals RHJ, Brouns SJJ. Distribution and Mechanism of the Type I CRISPR-Cas Systems. In: Barrangou R, van der Oost J, editors. CRISPR-Cas Systems: Springer; 2012. p 145-96.

[50] Makarova KS Haft DH Barrangou R Brouns SJ Charpentier E Horvath P Moineau S Mojica FJ Wolf YI Yakunin AF Evolution and classification of the CRISPR-Cas systems. Nat Rev Microbiol 2011; 9:467-477 10.1038/nrmicro2577 21552286 PMC3380444

[51] Mobley HL Hausinger RP Microbial ureases: significance, regulation, and molecular characterization. Microbiol Rev 1989; 53:85-108 2651866 10.1128/mr.53.1.85-108.1989 PMC372718

[52] Auch AF von Jan M Klenk HP Goker M Digital DNA-DNA hybridization for microbial species delineation by means of genome-to-genome sequence comparison. Stand Genomic Sci 2010; 2:117-134 10.4056/sigs.531120 21304684 PMC3035253

[53] Wayne LG Brenner DJ Colwell RR Grimont PAD Kandler O Krichevsky MI Moore LH Moore WEC Murray RGE Stackebrandt E Report of the Ad-Hoc-Committee on Reconciliation of Approaches to Bacterial Systematics. Int J Syst Bacteriol 1987; 37:463-464 10.1099/00207713-37-4-463

[54] Richter M Rossello-Mora R Shifting the genomic gold standard for the prokaryotic species definition. Proc Natl Acad Sci USA 2009; 106:19126-19131 10.1073/pnas.0906412106 19855009 PMC2776425

[55] In: Lapage SP, Sneath PHA, Lessel EF, Skerman VBD, Seeliger HPR, Clark WA, editors. International Code of Nomenclature of Bacteria: Bacteriological Code, 1990 Revision. Washington (DC)1992. 21089234

